# Trios—promising *in silico* biomarkers for differentiating the effect of disease on the human microbiome network

**DOI:** 10.1038/s41598-017-12959-3

**Published:** 2017-10-16

**Authors:** Zhanshan (Sam) Ma, Dandan Ye

**Affiliations:** Computational Biology and Medical Ecology Lab, State Key Lab of Genetic Resources and Evolution, Kunming Institute of Zoology, The Chinese Academy of Sciences, Kunming, 650223 China

## Abstract

Recent advances in the HMP (human microbiome project) research have revealed profound implications of the human microbiome to our health and diseases. We postulated that there should be distinctive features associated with healthy and/or diseased microbiome networks. Following Occam’s razor principle, we further hypothesized that triangle motifs or trios, arguably the simplest motif in a complex network of the human microbiome, should be sufficient to detect changes that occurred in the diseased microbiome. Here we test our hypothesis with six HMP datasets that cover five major human microbiome sites (gut, lung, oral, skin, and vaginal). The tests confirm our hypothesis and demonstrate that the trios involving the special nodes (e.g., most abundant OTU or MAO, and most dominant OTU or MDO, etc.) and interactions types (positive vs. negative) can be a powerful tool to differentiate between healthy and diseased microbiome samples. Our findings suggest that 12 kinds of trios (especially, dominantly inhibitive trio with mixed strategy, dominantly inhibitive trio with pure strategy, and fully facilitative strategy) may be utilized as in silico biomarkers for detecting disease-associated changes in the human microbiome, and may play an important role in personalized precision diagnosis of the human microbiome associated diseases.

## Introduction

With the rapid advances in the human microbiome research, it becomes increasingly important to detect and quantify the changes occurring in the human microbiome, especially the changes in the microbiome associated with disease. Differentiating between the healthy microbiome (sampled from healthy individuals) and diseased microbiome (sampled from individuals with microbiome associated disease such as bacterial vaginosis) is essentially a problem of measuring the dissimilarity between two microbial communities. Naturally, *diversity analysis* with traditional biodiversity measures such as species richness, Shannon information entropy (Shannon index), Simpson’s index, have been playing a dominant role in the field of comparing the human microbiome across space and time, as well as between healthy and diseased samples (HMP Consortium 2012a, 2012b, Lozupone *et al*. 2012)^1–3^. While diversity measures such as Shannon index are certainly useful for measuring the dissimilarity between communities, and indeed they have been applied to characterize microbial community in nearly every 16s-rRNA sequencing based microbiome study (*e.g*., Abusleme *et al*. 2013, Fodor *et al*. 2012, Srinivasan *et al*. 2012, Kong *et al*. 2012, McHardy *et al*. 2013)^4–8^, the diversity analysis is not without shortcomings. One inherent issue associated with all diversity measures is that they ignore the *interactions* between species, and they are simply some incarnations of species abundance distributions (SAD) in the community. Consequently, diversity analysis cannot fully account for the contributions of species interactions in the microbiome.

Although still not widely applied to the study of human microbiome, network analysis has been widely applied to other fields of computational biology and bioinformatics, such as gene regulatory and signal transduction networks, protein interaction networks, metabolic networks, phylogenetic networks, and ecological networks (Junker & Schreiber 2008)^9^. Indeed, network analysis, which considers both species abundance and their interactions (links), has been anticipated to remedy or even solve the issues associated with traditional diversity measures. In the field of human microbiome research, Faust and his collaborators (Faust & Raes 2012, Faust *et al*. 2012, 2015)^10–12^ performed extensive pioneering works. Since their works (Faust & Raes 2012, Faust *et al*. 2012, 2015)^10–12^, quite a few applications of network analysis in the human microbiome have been reported (*e.g*., Barberán *et al*. 2014, Chow *et al*. 2014, de Menezes *et al*. 2015, Duran-pinedo *et al*. 2011, Fernandez *et al*. 2015, Imangaliyev *et al*. 2015, Ma *et al*. 2015, 2016, Hunt *et al*. 2015, Ramayo-Caldas *et al*. 2016)^13–22^. Nevertheless, primarily due to the limitation with the existing microbiome datasets—almost all available microbiome datasets that can be utilized to build microbiome network models are species (OTU) abundance data from 16s rRNA sequencing technology, most of the successful applications are *ad hoc*. Accordingly, the standard *correlation network analysis* technique could only demonstrate limited power of network analysis in the study of human microbiome. In such applications to human microbiome research, the main results from standard correlation network analysis are a suite of network properties, including a series of simple network motifs (Shannon *et al*. 2003, Csardi & Nepusz 2006, Junker & Schreiber 2008)^9,23,24^, which often fail to produce conclusive, and occasionally may even generate conflicting evidence for differentiating between healthy and diseased microbiomes. We argue that the failure is due to some implicit assumptions of standard correlation networks (*e.g*., Shannon *et al*. 2003, Csardi & Nepusz 2006, Junker & Schreiber 2008)^9,23,24^. Correlation networks assume that nodes are ‘homogenous’ other than the heterogeneity in their correlation levels with immediate neighbors. For instance, in the case of human microbiome correlation networks, nodes (OTUs) are homogenous except for their abundances, which determine their correlation levels with their immediate neighbors in the network. Due to this implicit assumption, nodes lost their ‘identities’ other than some special roles such as hubs, identified by network properties. In the case of human microbiome network, nodes of *commensalists, facilitators, and* opportunistic pathogens may have very different functional roles, but the computation of network properties in standard correlation networks and motif detection do not even consider the difference between positive and negative links. The former may include facilitators, and the latter may be suppressed by beneficial microbes in the human microbiome. It is obvious that positive (facilitative) and negative (inhibitive) interactions may have very different biomedical implications. On the other hand, those implications associated with the interaction modes may not be reflected by the existing definition of correlation network properties and or motifs. Hence, identifying new network properties and/or network motifs that can capture the heterogeneities of node roles such as MAO (most abundant OTU) or hubs, MDO (most dominant OTU)^25^, as well as interaction modes (positive *vs*. negative) should remedy the insufficiency of standard correlation networks in analyzing the microbiome network, and offer potentially powerful tool for differentiating between healthy and diseased microbiomes.

In the present study, we propose to define/detect simple triangle motifs with a three-level hierarchical scheme that consider node role, interaction type (+ or –) and the combination of the role and type. Following Occam’s razor principle, we hypothesized that those *trios*, arguably the simplest motif in the OTU correlation network of the human microbiome, should be sufficient to detect significant changes occurred in the OTU correlation network of human microbiomes such as those impacted by or associated with diseases. The arguments supporting our hypothesis include: (*i*) our trios inherit the advantage of network analysis and therefore are able to overcome the disadvantage of *community diversity analysis*, which ignores the interaction between OTUs, (*ii*) our trios are advantageous over the *standard correlation network analysis* by considering the node role and interaction type, and should be more sensitive to the changes than the standard properties and motifs of correlation networks. As to the reason why we do not search for more complex motifs is due to the reality that finding arbitrary size motifs is a computationally NP-hard problem (Betzler *et al*. 2011, Tran *et al*. 2014)^26,27^, which, in a layman’s interpretation, implies that the computational time for finding all motifs of arbitrary size may be astronomical numbers even with the fastest computers humans have built. Therefore, if simple trio motifs, which are computationally lightweight to detect, can fulfill the mission to differentiate the microbiomes impacted by significant disturbances such as disease, complex and expensive computations become unnecessary.

In existing literature, our work is similar to the *triad*, which is a sub-graph consisting of three nodes and possible lines between the nodes, in social network analysis (O’Malley & Marsden 2008, Kitts & Huang 2010)^28,29^. However, the triads in social network analysis are directional, and a total of 16 triads were defined to describe directed interactions between three individuals. In our trio definitions, although our interactions (links) are directionless due to the nature of correlation network, we take into account the role of nodes (*e.g*., MAO) and the type of interactions (positive *vs*. negative). In addition, we consider trios with MAO (most abundant OTU) *handle*, *i.e*., the MAO is connected to a trio with possibly one, two, or three links (*i.e*., the *handle*). We consider this type of ‘trios,’ which consists of 4 nodes actually and, strictly speaking, should be termed “*quartos*,” because we found that the MAO may have far reaching effect on the whole structure of a trio beyond the effect on its immediate (directly connected) neighbor nodes. With a three-level classification scheme of *node role*, *interaction type*, and their *combinations* (specifically, the existence/absence of MAO handle or MAO, the positive *vs*. negative interaction, as well as their permutations), a total of 19 trios, including 10 *without MAO handle*, and 9 *with MAO handle*. The former class was further classified into 10 types based on the existence of MAO in the trio, as well as permutation of positive and negative links, and the latter class was further classified into 9 types based on the number of links to MAO (1, 2, or 3) as well as the permutation of positive and negative links. We developed the trios-finder program (TFP) in Perl for detecting the trios and provided the source code in the Supplementary information. We demonstrate the concept and design of using the trios as potential *in silico* biomarkers, as well as their implementation with the TFP software, with six HMP datasets that cover microbiomes and associated diseases from five major body sites (gut, lung, oral, skin, vaginal).

## The Method—Definitions and Computational Searching for Trios

### Defining the trios

Formally, with the three-level classification scheme, we distinguish two *classes*, five *categories*, and 19 *types* of trios at the first, secondary, and tertiary level, respectively (Table 1). At the primary level of the classification, we distinguish two *classes*, the *trios without* MAO *handle* and *trios with* MAO *handle*. At the secondary level, the first class refers to the triangle motif that is not connected with an *external* MAO ‘*handle*’ and is further distinguished as two *categories*: *trios with* MAO (*i.e*., MAO is part of the trio) and *trios without* MAO (*i.e*., MAO is not part of the trio). The second class refers to the triangle motif that is connected with an *external* MAO and is further distinguished as three *categories*: *single-link* MAO *handle*, *double-link* MAO *handle*, and *triple-link* MAO *handle*, with one, two and three links to the node of MAO handle, respectively.

**Table 1:** The 19 types of trios generated from a three-level classification scheme.

The *class* of “Trios without MAO Handle”	The *category* of “Trios with MAO”									The *category* of “Trios without MAO”				
	+ - - Type-1		∑	+ + - Type-2		∑	-	+	∑	-	+	+	+	∑
							-	+		-	-	+	+	
							- Type-3	+ Type-4		-	-	-	+	
.														
The *class* of “Trios with MAO Handle”	The *category* of “Single-Link MAO” (SLM Trio)				The *category* of “Double-Link MAO” (DLM Trio)					The *category* of “Triple-Link MAO” (TLM Trio)				
	− SLM–	+ SLM+	∑		−	+	+	∑		−	+	+	+	∑
					−	−	+			−	-	+	+	
										−	−	-	+	

At the tertiary level classification, each of the five *secondary* level categories (*trios without* MAO *handle, trios with* MAO *handle, single-link* MAO *handle*, *double-link* MAO *handle*, and *triple-link* MAO *handle*) is further classified based on the signs (+ or −) of the interactions (correlations) *within* the trio or *between* the trio and handle. Detailed classification of the 19 trio *types* at the tertiary level, generated from the above-described three-level classification scheme is presented in the following Table 1. Among 19 trio types, four types in the category of *trios without* MAO are nothing particular and are detected in existing network analysis software packages such as Cytoscape (Shannon *et al*. 2003)^23^ and iGraph (Csardi & Nepusz 2006)^24^. To the best of our knowledge, the other 15 trios have not been investigated in the existing literature. Our focus will be centered on those 15 special trios.

As it is demonstrated below, even the 15 special trios are not created equal, and some of the theoretically possible triangle motifs are not detected in our datasets and may even be ‘prohibited.’ Some of the trios, especially the six types of trios in the category of *trios with* MAO were named as Type-1A, Type-1B, Type-2A, Type-2B, Type-3, and Type-4, respectively. These six types are identified at the tertiary level classification; the sign (+ or −) of interaction in trios in the microbiome network is considered. For example, the difference between Type-1A and Type-1B lies in the signs of two links connected with the MAO, *i.e*., (+ −) in Type-1A and (− −) in Type-1B.

### Computational procedures

Our computational procedures for detecting the trios (triangle motifs) consist of the following four major steps: (*i*) computing OTU correlation coefficients (using either Spearman’s or Pearson’s definitions), (*ii*) filtering out false correlations with FDR (false discovery rate) adjustment, (*iii*) constructing the OTU (or species) correlation (interaction) networks with standard network analysis software packages such as Cytoscape (Shannon *et al*. 2003)^23^ or iGraph (Csardi & Nepusz 2006)^24^; and (*iv*) detecting the trios with home-made trio finder program (TFP) program, supplied in the online Supplementary document. The first two steps were actually implemented in a R-script CCFDR.r (Correlation Computing with False Discovery Rate) we provided in the online Supplementary materials. The R-script (CCFDR.r) calls the function “rcorr” from existing R-package Hmisc (https://cran.r-project.org/web/packages/Hmisc/) and the function “multiple.correction” from existing R-package EMA (https://cran.r-project.org/web/packages/EMA/index.html) to compute the correlation coefficients and filter out false correlations, respectively. The output from the CCFDR.r, *i.e*., correlation computing adjusted with FDR control, is feed into our Perl program TFP.pl, which completes the task of seeking and counting the various trio types. The following flowchart shows the computational procedures, and we further elaborate the possible issues involved in the procedures below.

A flowchart showing the four steps for implementing the trio-finding process:

**Step (i):** Compute the OTU (species) correlation coefficients with Spearman’s or Pearson’s definitions.

**Step (ii):** Filter out false correlation with FDR (false discovery rate) control with our CCFDR R-script.

**Step (iii):** Construct the OTU (or species) correlation (interaction) networks with standard network analysis software packages such as Cytoscape (Shannon et al. 2003)^23^ or iGraph (Csardi & Nepusz 2006)^24^. This step can be omitted if no network graphs are output.

**Step (iv):** Detecting the trios with our trio finder program (TFP.pl) (see the Supplement).

To construct SIN, we recommend using Spearman’s rank coefficient or occasionally Pearson’s correlation coefficient as demonstrated in Junker & Schreiber (2008)^9^, Ma *et al*. (2015, 2016)^20,21^.

However, to utilize the correlation coefficients for constructing the species or OTU correlation networks, there are two issues that should be addressed first: one is the choice of either the *relative abundance* or *actual OTU reads* and another is to filter out the false correlations in the raw correlation coefficient values in consideration of the rising risk of false correlations from multiple testing (*i.e*., simultaneously testing multiple null hypotheses or the significance of multiple correlation coefficients) with sequence data^30–32^. Both steps are usually necessary to ensure proper construction of the underlying OTU (species) correlation networks (SCN), also known as species interaction networks (SIN) as often termed in macro-ecology.

Regarding the utilization of OTU reads for computing the correlation coefficients, our recommendation is that, when the numbers of sequence reads from different samples are approximately equal, the OTU reads can be utilized directly to compute the correlation coefficients; when the numbers of sequences reads from different samples are significantly different, the *relative* abundances should be utilized instead. The usage of OTU reads directly has an advantage over the relative abundance since the former can avoid the potential error from decimal conversion in calculating the relative abundance (*i.e*., the OTU reads for a particular OTU or species divided by the total number of reads of all OTUs in the sample). Our pre-experiment tests found that, although both *relative abundance* and *absolute abundance* (raw OTU reads) may produce different results when the numbers of sequencing reads across samples are different, the trend of trios is rather robust. In this study, we use the relative abundance (*i.e*., the reads of a particular OTU in a sample divided by the total reads in the sample) to be fail-safe. Alternatively, if one does not wish to use relative abundance, sub-sampling (*i.e*., randomly choose the same number of reads from each sample, *e.g*., 5000 reads from each sample) may be utilized to deal with the issues associated with unbalanced sample sizes.

To deal with the rising chances that some tests will tend to pass falsely when simultaneously testing multiple null hypotheses (*i.e*., the significance of many correlation relationships) in 16s-rRNA sequence data, we suggest correcting the *p*-values associated with the correlation coefficients (from either Spearman’s or Pearson’s methods) with the FDR-BH algorithm (Benjamini-Hochberg standard false discovery rate correction) (Benjamini and Hochberg’s 1995)^30^. The procedures with the FDR control have been implemented in several R packages, and we choose to use the R-package EMA, which implemented FDR-BH algorithm by Servant *et al*. (https://cran.r-project.org/web/packages/EMA/index.html)^31^^.^ Specifically, we called the “multiple.correction” function from the EMA package in our own R-script “CCFDR.r”, which also called another function rcorr from the R-package Hmisc (https://cran.r-project.org/web/packages/Hmisc/) to compute the Spearman’s or Pearson’s correlation coefficients. Our R-script “CCFDR.r” essentially implemented the first two steps in previous flowchart and its output is feed into our Perl program TFP.pl (Trios-Finder Program), which completes the trios-finding function outlined in step 4 in the previous flowchart. Both CCFDR.r and TFP.pl are supplied in the online Supplementary materials.

In summary, after dealing with the above-described two potential issues with our “CCFDR.r” R-script (*i.e*., eliminating the side effect of unbalanced of sample sizes and filtering out false correlations) we use the remaining correlation relationships (*i.e*., Spearman’s rank correlation coefficient (ρ) in this study) with a threshold of p ≤ 0.05 to build OTU correlation networks for the healthy and diseased microbiome samples, respectively. From the OTU correlation networks, the trios defined in Table 1 are sought out and counted with our homemade Perl-program TFP.pl.

In this article, we present our methodology and hypothesis based on the trios that are associated with the MAO (most abundant OTU) in the microbial species interaction network to simplify the presentation. It is noted that our methodology presented here regarding the special node can be readily extended to other nodes in SIN with some special biomedical or computational implications. We have also applied the MDO (most dominant OTU) and hub associated trios elsewhere (Ma & Ellison 2017a)^25^ with the same computational procedures presented here, but the detailed approaches are only reported in this article.

To compare the distribution of the above-defined trios in the healthy and diseased microbiome samples, we define RDHT (the ratio of disease to healthy trios), the number of a particular trio type in the disease treatment divided by the number of the same trio type in the healthy counterpart. Nevertheless, caution should be taken to use RDHT for diagnostic purpose since the magnitude of different trio types may be different. In addition, the identity of trio members may also be of critical biomedical significance.

## Test Results and Discussion

### Test dataset description

Largely following the sampling scheme of NIH human microbiome project, we selected six datasets that represent the microbiomes sampled from five major body sites (gut, lung, oral, skin, and vagina). Except for gut that is represented by two datasets (HIV and IBD), each microbiome site is represented by one dataset, with six datasets in total. A brief description on the six datasets is summarized in Table 2, and detailed information on individual dataset is referred to the original publication noted in Table 2.

**Table 2:** Datasets utilized to develop and test the TFP (trio finder program)

*Microbiome site*	*Associated Disease*	Treatments*	*Reference*
Gut	IBD (Inflammatory Bowel Disease)	Crohn’s disease (CD, 18), Ulcerative colitis (UC: 38, 18), Healthy (18)	Agouridis *et al*. (2011)
	HIV	HIV Negative (20), ART (20), Non-ART (20)	McHardy *et al*. (2013)
Lung	Cystic Fibrosis (CF)	Exacerbation (25) *vs*. End of Treatment (25)	Fodor *et al*. (2012)
Oral	Periodontitis	Healthy (17), Periodontitis with bleeding (PB) (22), Periodontitis without bleeding (PnB)(22)	Abusleme *et al*. (2013)
Skin	Atopic Dermatitis (AD)	AD (36, 22) *vs*. Healthy (22)	Kong *et al*. (2012)
Vaginal	Bacterial Vaginosis (BV)	BV (117) *vs*. healthy (103)	Srinivasan *et al*. (2012)

.*The numbers parenthesized are the sample sizes, *i.e*., the number of individuals sampled in the original study or that used for constructing species correlation network. When there are two numbers (sample sizes) in the parentheses, the first number is the sample size of the original study, and the second number is the actual sample size we used to reconstruct the species correlation network in this article. The reason to use a different sample size from the original sample size is to avoid the influence of unbalanced treatments—the difference between treatments in the sample size is large enough to influence the results of network analysis. For example, in the case of IBD, the number of UC samples is 38, which is significantly more than the numbers of samples in the two other treatments (both are 18). To avoid the side effect of unbalanced treatments, 18 samples were drawn out of 38 samples and the sampling process was repeated for 50 times. From each sampling process, a network was reconstructed and the network properties including the numbers of trios were computed. The averages from the 50 sampling operations were taken as the final result for the sampled treatment. Two treatments, *i.e*., UC in IBD case, AD in Skin case were sampled to deal with the issue of unbalanced treatments.

For each of the six case studies representing the five major microbiome sites, we constructed separate species interaction networks (SIN) with the 16s-rRNA sequence dataset from each treatment in the six case studies. For example, with BV dataset (Srinivasan *et al*.)^6^, we built two SINs: one with the 16s-rRNA microbiome samples from BV group and another with the samples from healthy group. We followed the 4-step computational procedure described in the previous section and built 15 networks in total for the 15 treatments of the healthy and diseased microbiome groups, covering five major microbiome sites (gut, lung, oral, skin, and vaginal) and representing several diseases including HIV-infection, inflammatory bowel disease (IBD), periodontitis, cystic fibrosis (CF), Atopic Dermatitis (AD), and bacterial vaginosis (BV), as detailed in previous Table 2.

After getting respective SIN for each of the 15 treatments of the six case studies, we utilized our homemade trio finder program (TFP.pl) program, supplied in the Supplementary document, to compute the 19 triangle motifs or trio types defined in previous Table 1. The results from TFP computing are listed in the following Table 3 for the class of “*trios without MAO handle*” and Table 4 for the class of “*trios with MAO handle*,” respectively.

**Table 3:** The number of various *trios* in the class of “*Trios without MAO handle*” in the SIN.

Microbiomes & Associated Disease Treatments		Trios with MAO									Trios without MAO				
		+ - - Type-1		∑	+ + - Type−2		∑	Type-3 −	Type-4 +	∑	−	+	+	+	∑
								–	+		−	−	+	+	
								–	+		−	−	−	+	
**Gut**	HIV-Negative	36	14	50	0	0	0	0	86	136	0	365	0	2127549	2127914
	HIV-ART	11	22	33	0	0	0	0	4	37	0	125	0	1683975	1684100
	HIV-NonART	3	0	3	0	0	0	0	110	113	0	43	0	1876511	1876554
	IBD-Healthy	0	0	0	0	0	0	0	2	2	0	0	0	278775	278775
	IBD-CD	0	0	0	0	0	0	0	5	5	0	0	0	86738	86738
	IBD-UC	1	0	1	0	0	0	0	34	35	0	11	0	280215	280226
**Lung**	End of treatment	0	0	0	0	0	0	0	0	0	0	0	0	562	562
	Exacerbation	1	0	1	0	0	0	0	0	1	0	0	0	1055	1055
**Oral**	Healthy	0	21	21	0	0	0	0	0	21	0	27	0	42155	42182
	PB	0	0	0	0	0	0	0	0	0	0	3	0	9783	9786
	PnB	0	0	0	0	0	0	0	0	0	0	0	0	11607	11607
**Skin**	Healthy	0	0	0	0	0	0	0	0	0	0	0	0	0	0
	AD	0	30	30	0	0	0	0	0	30	0	0	0	65	65
**Vaginal**	Healthy	0	0	0	0	0	0	0	0	0	0	1	0	5327	5328
	BV	6	17	23	0	0	0	0	2	25	0	39	0	428	467

**Table 4:** The number of various *trios* in the class of “*Trios with MAO handle*” in the SIN.

Microbiomes & Associated Disease Treatments		Single-Link MAO			Double-Link MAO				Triple-Link MAO				
		-	+	∑	-	+	+	∑	-	+	+	+	∑
					-	-	+		-	-	+	+	
									-	-	-	+	
Gut	HIV-Negative	1029	311	1340	207	123	286	616	8	37	76	239	360
	HIV-ART	434	21	455	171	17	1	189	24	13	3	1	41
	HIV-NonART	1	1381	1382	0	2	260	262	0	0	2	240	242
	IBD-Healthy	0	1	1	0	0	0	0	0	0	0	0	0
	IBD-CD	0	25	25	0	0	4	4	0	0	0	1	1
	IBD-UC	8	633	641	0	1	123	124	0	0	1	71	72
Lung	End of treatment	0	0	0	0	0	0	0	0	0	0	0	0
	Exacerbation	0	0	0	0	0	0	0	0	0	0	0	0
Oral	Healthy	485	0	485	192	0	0	192	27	0	0	0	27
	PB	0	2	2	0	0	0	0	0	0	0	0	0
	PnB	0	0	0	0	0	0	0	0	0	0	0	0
Skin	Healthy	0	0	0	0	0	0	0	0	0	0	0	0
	AD	2	0	2	10	0	0	10	53	0	0	0	53
Vaginal	Healthy	1	0	1	0	0	0	0	0	0	0	0	0
	BV	60	24	84	31	14	3	48	14	6	1	0	21

### The class of “Trios without MAO handle”

In consideration of the existence or absence of MAO handle connected with trios, the 19 triangle motifs are classified into two *classes* (explained in Table 1) *trios without MAO handle* (upper section in Table 1 and discussed in this sub-section further) and *trios with MAO handle* (bottom section in Table 1 and discussed in the next sub-section).

As displayed in Table 3, the class of *trios without MAO handle* is further distinguished as two categories: *trios with MAO* and *trios without MAO*. The category of *trios without MAO*, including four types that differ from each other by the signs of trio links [(– – –), (+ – –), (+ + –), (+ + +)], although listed for comparative purpose, may be less important than the category of *trios with MAO* for the following reason: because MAO is not involved apparently, the number of trios in this category may be too many to focus on for further etiological studies in practice.

Given the particular significance of the *category* of *trios with MAO*, we further distinguish four *types* (Type 1–4) at the tertiary level classification, depending on the signs (+ or –) of the trio links. Due to the special role of MAO, we further distinguish two sub-types for Type-1 (Type-1A & Type-1B) and Type-2 (Type-2A & Type 2B), respectively, at the tertiary level by noting the ‘position’ of MAO in the trio (see Table 1). This classification results in the six *types* in the category of *trios with MAO*, *i.e*., Type-1A, Type-1B, Type-2A, Type-2B, Type-3, and Type-4 (also see previous Table 1).

Table 3 shows that theoretically possible Type-2 and Type-3 were not detected in our case studies. The counterpart types in the category of *trios without MAO* were not detected either. Actually, the apparent prohibition of both Type-2 and Type-3 is not difficult to explain by their internal interactions. Type-2, which has three links with (+ + –) interaction relationships, may be hard to sustain because a third link of negative (–) interaction would be ‘coerced’ to follow the ‘mainstream norm’ of two other collaborative relationships. Similarly, a trio consisting of three totally opposing nodes is unlikely to sustain because they would most likely ‘destroy’ each other. Obviously, regardless whether or not MAO is involved, the arguments regarding (+ + –) and (– – –) hold; hence in both trios *with* and *without* MAO, these two patterns may not be sustainable.

We name Type-1 (+ – –) triangle motif as *dominantly inhibitive* trio given that negative interactions form majority in the system. In Type-1A sub-type, MAO takes a mixed strategy, collaborating with one and competing with another node in the trio. We term Type-1A as *dominantly inhibitive trio with mixed strategy*. In the Type-1B sub-type, MAO competes with both nodes in the trio simultaneously, and we term this type *dominantly inhibitive trio with pure strategy*. Among the six tested cases displayed in Table 3, in the cases of skin and oral, no Type-1A trio was detected; in the cases of IBD and lung, no Type-1B was detected. In the other cases, the RDHT of Type-1 ranged from 0 to infinity. That is, disease may raise or lower the number of Type-1 trios depending on the type of microbiome and its associated disease, possibly on other factors, and the difference can be exploited to detect the impact of diseases.

We name Type-4 (+ + +) triangle motif as *fully facilitative trio* given that positive interactions are the sole interaction in this type of trio system. It is also the most abundant triangle motif among the four types in the category of *trios with MAO*.

In summary, the results in Table 3 suggest that *dominantly inhibitive trio* (*i.e*., Type-1, including both Type-1A with *mixed strategy* and Type-1B with *pure strategy*) and *fully facilitative trio* (Type-4) possess the potential to act as *in silico* biomarker for differentiating the healthy and diseased microbiomes. As to the criteria for differentiating disease from healthy microbiome, we previously defined the ratio of disease to healthy trio (RDHT) as indicator of the changes, but actual application of the indicator is individual case specific, depending on the types of microbiome, disease, and possibly other factors. In fact, the taxonomic identities and biological characteristics (such as anaerobes or opportunistic pathogens) of trio nodes should play a rather important role in deciphering the mechanism of specific trio formation as we demonstrate elsewhere.

### The class of “Trios with MAO handle”

In the class of trios *with MAO handle*, MAO is connected with the trio as a ‘handle’ rather than as a constituent node of the trio. In contrast with the previously discussed class of trios without MAO handle, there is no ‘prohibited’ trio in the *trios with MAO handle*. Therefore, all three categories (SLM, DLM, and TLM) including nine types (classified at the tertiary level by considering the link signs) are practically possible. Note that the trios in this class actually do not contain MAO because usually MAO is unique in microbiome network.

The results in Tables 5 and 6 suggest that the range of RDHT spans from zero to infinity. In a half of the cases (48%, or 13 out of 27 cases in the last three columns of Table 5), RDHT exceeded one, that is, diseases tend to raise the number of trios in the class of *trios with MAO handle*. Furthermore in 44% of the cases (12 out of 27), the RDHT exceeded 10, *i.e*., diseases caused more than 10 times increase in the number of trios; in 1/3 of the cases (9 out of 27), the RDHT reaches infinity, *i.e*., the trios occurred only in diseased microbiome networks. Given the striking differences in RDHT among different microbiome-disease treatments, we consider this class of trios also possesses the potential to act as *in silico* biomarker for assessing the effects of diseases on the human microbiome. Since it seems that the numbers of trios in this class are far greater than those in the class of *trios without MAO handle*, we believe that the previously identified *fully facilitative trios* and *dominantly inhibitive trios* may have an advantage over this class in exploring the mechanisms of disease effects. Another argument in support of our opinion is that the *fully facilitative trios* and *dominantly inhibitive trios* are simpler with three nodes only.

**Table 5:** The RDHT (ratio of disease to healthy trios) of category-specific total trios in the human microbiome computed from Tables 3 & 4.

Site	**RDHT** (Ratio of D/H Trios)	Trios with MAO	Trios without MAO	Single link with MAO	Double link with MAO	Triple link with MAO
Gut	HIV-ART/Negative	0.27	0.79	0.34	0.31	0.11
	HIV-NonART/Negative	0.83	0.88	1.03	0.43	0.67
	IBD-CD/IBD-Healthy	2.50	0.31	25.00	Infinite	Infinite
	IBD-UC/IBD-Healthy	17.50	1.01	641.00	Infinite	Infinite
Lung	Exacerbation/Treatment	Infinite	1.88	NA	NA	NA
Oral	PB/Healthy	0.00	0.23	0.00	0.00	0.00
	PnB/Healthy	0.00	0.28	0.00	0.00	0.00
Skin	AD/Healthy	Infinite	Infinite	Infinite	Infinite	Infinite
Vaginal	BV/Healthy	Infinite	0.09	84.00	Infinite	Infinite

**Table 6:** The RDHT (ratio of disease to healthy trios) of Type-1, 2, 3, 4 trios (*i.e*., Trios with MAO but no MAO handle) in the human microbiome computed from Table 3.

Site	**RDHT**	Type-1		Type-2		Type-3	Type-4
		Type-1A	Type-1B	Type-2A	Type-2B		
Gut	HIV-ART/Negative	0.31	1.57	NA	NA	NA	0.05
	HIV-NonART/Negative	0.08	0.00	NA	NA	NA	1.28
	IBD-CD/IBD-Healthy	Infinite	NA	NA	NA	NA	2.50
	IBD-UC/IBD-Healthy	Infinite	NA	NA	NA	NA	17.00
Lung	Exacerbation/Treatment	Infinite	NA	NA	NA	NA	NA
Oral	PB/Healthy	NA	0.00	NA	NA	NA	NA
	PnB/Healthy	NA	0.00	NA	NA	NA	NA
Skin	AD/Healthy	NA	Infinite	NA	NA	NA	NA
Vaginal	BV/Healthy	Infinite	Infinite	NA	NA	NA	Infinite

### General patterns of the trio differences between the healthy and diseased microbiomes

In the following, we further look into general patterns by cataloging the 19 trios into *five categories* and summing up the total trios of each category in Table 5. In Table 5, besides listing the microbiome sites and healthy/disease treatments in the first two columns, the remaining five columns display the respective ratios of the trios in diseased network to those in healthy network for each of the *five* categories, *i.e*., RDHT for: trios with MAO, trios without MAO, single-link with MAO (SLM), double-link with MAO (DLM) and triple-link with MAO (TLM), respectively. That is, for each category, we define and compute the ratio of diseased *to* healthy trios (RDHT). In the ideal scenario when disease has no impact on the microbiome, the RDHT should be 1. If the ratio is larger than 1, then it indicates that the disease may raise the number of trios in the specific category; *vice versa*, it indicates that the disease may lower the number of trios if the RDHT is smaller than 1.

Table 5 shows that in approximately 47% cases (21 out of 45) disease caused a decrease in the number of trios. Specifically, the decline of trios occurred mostly in two diseases: HIV and periodontitis. The RDHT values range from zero to infinity; the occurrence of zero or near zero (≤0.1) counts to 9, and that of infinity reaches 29% (13 out of 45). The number of RDHT exceeding 10 (*i.e*., disease caused more than 10 times of increase in the trios) approaches to 38% (17 out of 45). In these cases, disease led to a significant increase in the number of trios.

More interesting insights can be found by looking into the third level classification—considering the sign (+ , –) of interactions (links) in the trios, as well as the ‘position’ of signs (see Table 1). Table 6 summarized the RDHT of Type-1, 2, 3, 4 trios from the information presented in Tables 3 & 4 to further reveal patterns and trends embedded in the six trios that are associated with MAO but without a MAO handle (see Table 1). Of course, the undetected Type-2 and Type-3 appear to be “prohibited” in our case studies as explained previously.

Among the six cases we analyzed, except for the CF-lung case, which we cannot draw a definite conclusion due to data insufficiency, there were three cases (IBD-gut, AD-skin, and BV-vaginal) that displayed disease-up-regulated trios trend, and two cases (HIV-gut and periodontitis-oral) displayed disease-down-regulated trend. Although further accumulating test cases is certainly meaningful, this splitting trend of the up or down of trios does not affect the testing of our primary hypothesis—whether or not the trios we defined can differentiate between healthy and diseased microbiomes. This is because the validity of our hypothesis hinges on the *level of difference* or *gap* in the trio numbers (*i.e*., RDHT) rather than on the sign or direction of the difference (rise or decline). Indeed, we believe that the variable sign of the difference among microbiomes may simply be a biomedical reality.

Finally, we suggest that, among 19 types of trios we initially propose to test, 12 are indeed promising as *in silico* biomarkers. The six trio types we excluded are the four types in the category of “*trios without MAO*”, and three types (Type-2A, Type-2B, & Type-3) in the category of “trios with MAO”. The reason they are excluded is either because they are either too abundant (to be indicative) or too rare (not detected) in both healthy and diseased samples, to be indicative. Indeed, the entire category of “trios without MAO” is excluded, not only because they are too abundant to be indicative, but also because they lack special node (in this study, the MAO). We demonstrated that the following 12 types or categories are the most promising: Type 1A (*dominantly inhibitive trio with mixed strategy*), Type 1B (*dominantly inhibitive trio with pure strategy*), Type-4 (*fully facilitative*), SLM (including 2 types), DLM (3 types), and TLM (4 types). We particularly favor the first three types, and give them the special names in particular in consideration that they are simpler and less abundant (in general) than the four-nodes SLM, DLM, and TLM. This may give them an advantage in further studying their etiological implications experimentally. As mentioned previously, two further improvements can be made to reveal potentially more meaningful biomedical insights. One is to look up the taxonomic identities or biomedical characteristics such as the trios of anaerobes, and another is to replace the MAO with other special network nodes such as MDO (most dominant OTU) or hub nodes. We will demonstrate these additional improvements elsewhere.

### Data accessibility

The datasets utilized in this study are available in the original studies cited in Table 2. The study does not involve any experiments involving humans and/or animals.

## Electronic supplementary material

Supplementary Data

## References

[1] HMP Consortium (Human Microbiome Project Consortium) (2012). A framework for human microbiome research. Nature.

[2] HMP Consortium (Human Microbiome Project Consortium) (2012). Structure, function and diversity of the healthy human microbiome. Nature.

[3] Lozupone CA, Stombaugh JI, Gordon JI, Jansson JK, Knight R (2012). Diversity, stability and resilience of the human gut microbiota. Nature.

[4] Abusleme L (2013). The subgingival microbiome in health and periodontitis and its relationship with community biomass and inflammation. The ISME Journal.

[5] Fodor AA (2012). The Adult Cystic Fibrosis Airway Microbiota is Stable over Time and Infection Type, and Highly Resilient to Antibiotic Treatment of Exacerbations. Plos One.

[6] Srinivasan S (2012). Bacterial Communities in Women with Bacterial Vaginosis: High Resolution Phylogenetic Analyses Reveal Relationships of Microbiota to Clinical Criteria. PLoS ONE.

[7] Kong. HH (2012). Temporal shifts in the skin microbiome associated with disease flares and treatment in children with atopic dermatitis. Genome Res..

[8] McHardy IH (2013). HIV infection is associated with compositional and functional shifts in the rectal mucosal microbiota. Microbiome.

[9] Junker, B.H., Schreiber, F. Analysis of Biological Networks, Wiley-Interscience, N.J, USA (2008).

[10] Faust K, Raes J (2012). Microbial interactions: from networks to models. Nat Rev Micro.

[11] Faust K (2012). Microbial co-occurrence relationships in the human microbiome. PLoS Comput Biol.

[12] Faust K, Lahti L, Gonze D, de Vos WM, Raes J (2015). Metagenomics meets time series analysis: unraveling microbial community dynamics. Current Opinion in Microbiology.

[13] Barberán A, Casamayor EO, Fierer N (2014). The microbial contribution to macroecology. Frontiers in Microbiology.

[14] Chow CT, Kim DY, Sachdeva R, Caron DA, Fuhrman JA (2014). Top-down controls on bacterial community structure: microbial network analysis of bacteria, T4-lik viruses and protists. The ISME Journal.

[15] de Menezes AB (2015). Network analysis reveals that bacteria and fungi form modules that correlate independently with soil parameters. Environmental Microbiology.

[16] Duran-pinedo AE, Paster B, Teles R, Frias-Lopez J (2011). Correlation network analysis applied to complex biofilm communities. Plos ONE.

[17] Fernandez M, Riveros JD, Campos M, Mathee K, Narasimhan G (2015). Microbial “Social networks”. BMC Genomics.

[18] Imangaliyev S, Keijser B, Crielaar W, Tsivtsivadze T (2015). Personalized microbial network inference via co-regularized spectral clustering. Methods.

[19] Ma, Z. S. *et al*. A brief review on the ecological network analysis with applications in the emerging medical ecology. *Hydrocarbon and Lipid Microbiology Protocols*, Springer (2016).

[20] Ma ZS, Guan Q, Ye C (2015). Network analysis suggests a potentially ‘evil’ alliance of opportunistic pathogens inhibited by a cooperative network in human milk bacterial communities. Scientific Reports.

[21] Hunt, D. E., Ward, C. S. A network-based approach to disturbance transmission through microbial interactions. *Frontiers in Microbiology*, **6**, Article 1182 (2015). 10.3389/fmicb.2015.01182 PMC4621455 26579091

[22] Ramayo-Caldas, Y. *et al*. Phylogenetic network analysis applied to pig gut microbiota identifies an ecosystem structure linked with growth traits. *The ISME Journal* 1–5. (2016). 10.1038/ismej.2016.77 PMC5148198 27177190

[23] Shannon, P. *et al*. Cytoscape: a software environment for integrated models of biomolecular interaction networks. *Genome Res* **13**, (2003). 10.1101/gr.1239303 PMC403769 14597658

[24] Csardi, G. & Nepusz, T. The igraph software package for complex network research. InterJournal. *Complex Systems*: 1695. (2006).

[25] Ma Z. S., Ellison A. M. A new dominance concept and its application to diversity-stability analysis. http://adsabs.harvard.edu/cgi-bin/bib_query?arXiv:1703.08835 (2017a).

[26] Betzler N, Bevern R, Fellows MR, Komusiewicz C, Niedermeier R (2011). Parameterized Algorithmics for Finding Connected Motifs in BiologicalNetworks. IEEE/ACM Trans. On Computational Biology and Bioinformatics.

[27] Tran, N.T.L., Mohan, S., Xu, Z., Huang, C.H. Current innovations and future challenges of network motif detection. *Briefs in Bioinformatic*s, 10.1093/bib/bbu021, 1–29pp. (2014). 10.1093/bib/bbu021 24966356

[28] O’Malley AJ, Marsden PV (2014). The Analysis of Social Networks. Health Serv Outcomes Res Methodol..

[29] Kitts, J.A., Huang, J. “Triads.” Encyclopedia of Social Networks, George Barnett, Editor. New York: Sage Publications (2010).

[30] Benjamini Y, Y. Hochberg. (1995) Controlling the false discovery rate: a practical and powerful approach to multiple testing. *J. R. Statistic. Soc. B* **57**(1), 289–300 (1995).

[31] Servant, N *et al*. Package ‘EMA’: https://CRAN.R-project.org/package=EMA, Version 1.45 (2016)

[32] Nobel WS (2009). How does multiple testing correction work?. Nature Biotechnology.

[33] Agouridis AP, Elisaf M, Milionis HJ (2011). An overview of lipid abnormalities in patients with inflammatory bowel disease. Annals of Gastroenterology: Quarterly Publication of the Hellenic Society of Gastroenterology.

