# Supplementary material for: Trios—promising in silico biomarkers for differentiating the effect of disease on the human microbiome network
Source: Sci Rep. 2017 Oct 16;7:13259. doi: 10.1038/s41598-017-12959-3 (PMC5643543; doi:10.1038/s41598-017-12959-3)
Supplement: Supplementary file 1 — Supplementary Data [file 41598_2017_12959_MOESM1_ESM.zip › Trios-Code&Demo/Trios-Help File.pdf]

Supplementary materials for Ma & Ye (2017) Trios—promising in silico biomarkers for differentiating the effect of disease on the human microbiome network. *Scientific Reports*.

The Help file for the CCFDR.r and TFP.pl programs

CCFDR.r: **Correlation Computing with False Discovery Rate (FDR) Control**

(1) Program installation: The *R* version we used in this step is version 3.2.3. The R-script (CCFDR.r) requires the pre-installation of "Hmisc" and "EMA" packages for *R*. The script (CCFDR.r) and its input data folder (*e.g*., "data") should be located in the same folder.

(2) Input file
The input *file* is a data folder, named "data", and each file in this folder is an OTU table, an MxN matrix with elements representing the number of OTU reads for each OTU in the community, separated by "\t" (Tab) symbol, where M is the number of community samples, and N is the number of OTUs. The first row lists the OTU names, and the first column lists sample IDs or names.

(3) Usage
Copy the R-script (CCFDR.r) and input file folder (*e.g*., "data") to the same folder (*e.g*., trios-folder).
Enter the following command under Linux shell prompt:
$ Rscript CCFDR.r

(4) Output folder (SIN-network)
The output from the R-script CCFDR is stored in "SIN-network". Each file in this folder is actually a *network* file containing nodes and edges for building the species interaction network (SIN). In each SIN file, each network edge is represented by one line (with four columns) in the file. The first two columns represent for the two nodes of an edge, and the last two columns represent for the correlation coefficients and corresponding corrected *p*-values.

TMF.pl: **Trio finder program (TFP) for finding and counting the trios**

(1) Program installation: Make sure Perl language environment (Version 5.1 or later) is installed on your system. Copy TMF.pl, input folder (*e.g*., "SIN-network" in our case) and MAO (most abundant species) file (*e.g*. MAO.txt) to the same folder (*e.g.,* trios).

(2) Input file
One input file (folder) for the TMF.pl program is the previously mentioned output file (folder) from CCFDR.r R-script (*e.g.,* "SIN-network" in our case). Another input file for TMF.pl is a file containing the most abundant species (MAO) for each network (*e.g*. MAO.txt). In this input file, each row consists of three items, the ID of network, the MAO species and corresponding abundance of the network, respectively, separated by "\t" (Tab) symbol.

(3) Usage
Copy the Perl program (TMF.pl), input folder (*e.g*., "SIN-Network") and MAO file (*e.g*. MAO.txt) to the same folder (*e.g.,* trios). Enter the following command under Linux shell prompt:
$ perl TFP.pl

(4) Output file (Trios.txt)

There are 27 columns in the output file and the explanations for each column is described in the following table. The first row in the output file is the name of the trios, and the other rows are the count (number) of each trio type.

Trios	Note
MAO + – – (Type-1A)	Trios with MAO, Type-1A
MAO – + – (Type-1B)	Trios with MAO, Type-1B
MAO (Type-1)	Sum of Type-1A and Type-1B
MAO + + – (Type-2A)	Trios with MAO, Type-2A
MAO + – + (Type-2B)	Trios with MAO, Type-2B
MAO (Type-2)	Sum of Type-2A and Type-2B
MAO – – – (Type-3)	Trios with MAO, Type-3
MAO + + + (Type-4)	Trios with MAO, Type-4
Trios with MAO (but without MAO handle)	Sum of Trios with MAO
– – –	Trios without MAO
– – +	
– + +	
+ + +	
Trios without MAO (without MAO handle)	Sum of Trios without MAO
SLM –	Single-Link MAO (SLM) (–)
SLM +	Single-Link MAO (SLM) (+)
SLM	Sum of SLM
DLM – –	Double-Link MAO (DLM) (––)
DLM – +	Double-Link MAO (DLM) (+–)
DLM++	Double-Link MAO (DLM) (++)
DLM	Sum of DLM
TLM – – –	Triple-Link MAO (TLM) (–––)
TLM – – +	Triple-Link MAO (TLM) (+––)
TLM – + +	Triple-Link MAO (TLM) (++–)
TLM + + +	Triple-Link MAO (TLM) (+++)
TLM	Sum of TLM
